# Case Report: Immune Checkpoint Inhibitors Successfully Controlled Asymptomatic Brain Metastasis in Esophageal Squamous Cell Carcinoma

**DOI:** 10.3389/fimmu.2022.746869

**Published:** 2022-03-01

**Authors:** Linlin Xiao, Chi Lin, Yueping Liu, Yajing Wu, Jun Wang

**Affiliations:** ^1^ Department of Radiation Oncology, The Fourth Hospital of Hebei Medical University, Shijiazhuang, China; ^2^ Department of Radiation Oncology, University of Nebraska Medical Center, Omaha, NE, United States; ^3^ Department of Pathology, The Fourth Hospital of Hebei Medical University, Shijiazhuang, China

**Keywords:** brain metastasis, esophageal carcinoma, PD-L1, immune checkpoint inhibitors, esophageal squamous cell carcinoma

## Abstract

**Background:**

Brain metastases are the most common cause of intracranial malignancy, often resulting in significant morbidity and mortality. Brain metastases from esophageal squamous cell carcinoma (ESCC) are relatively rare, with a rate of generally less than 2%.

**Case Report:**

In this article, we report a rare case of ESCC with asymptomatic brain metastasis. The combined positive score (CPS) of programmed cell death-ligand 1 (PD-L1) from the primary tumor was 2 by DAKO 22C3 and 3 by VENTANA SP263. The proportion of tumor infiltrating lymphocytes (TILs) was 1%. After receiving 15 cycles of immune checkpoint inhibitors (ICIs), the patient’s brain metastatic lesion had disappeared and was replaced by a local necrotic area. He retains good cognitive function with a stable disease at the primary site.

**Conclusions:**

This is the first to be reported in an ESCC patient whose brain metastatic lesion had a complete response to ICIs, which may provide supporting data for using ICIs as an option of treatment for ESCC patients with brain metastases.

## Introduction

Brain metastases are the most common cause of intracranial malignancy, often resulting in significant morbidity and mortality (1). Brain metastases usually occur in lung cancer, breast cancer and melanoma, which account for approximately 67-80% of patients. Brain metastases from esophageal carcinoma (EC) are relatively rare, with a rate of generally less than 2% (2–4).

Recently, immune checkpoint inhibitors (ICIs) in the treatment of patients with EC was in full swing. The KEYNOTE-181, ATTRACTION-3 and ESCORT studies had demonstrated that ICIs could improve the overall survival (OS) with a manageable toxicity profile in previously treated patients with advanced or metastatic EC, representing a standard second-line treatment option for these patients (5–7). As the low incidence of brain metastases in patients with EC, the three studies did not include patients with brain metastases and there is no study on ICIs for these patients at present. Here, we presented a case of asymptomatic brain metastasis from EC. The patient achieved intracranial complete response by the treatment of ICIs. Although this phenomenon had been reported in other cancers (8, 9), it is the first to be reported in EC, which may provide supporting data for using ICIs as one of options for management of brain metastases from esophageal cancer.

## Case Report

**Figure 1:**
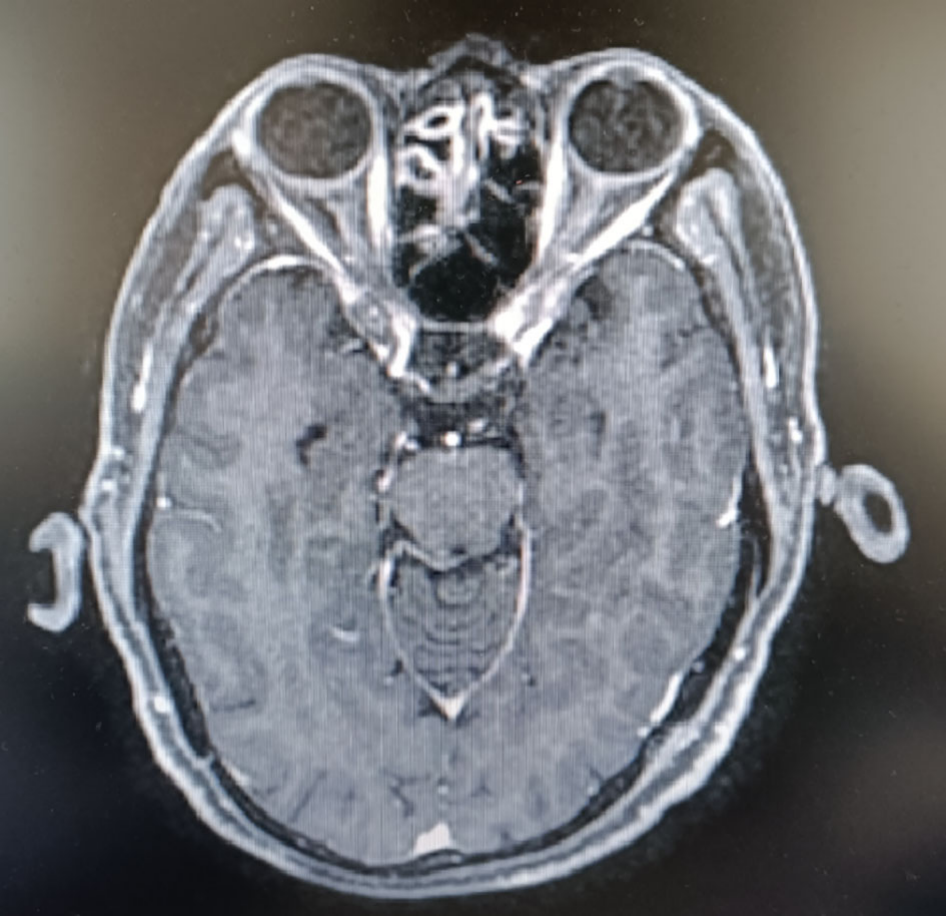
The baseline brain MRI of the patient.

A 66-year-old male patient presented himself to our oncological outpatient clinic with odynophagia in February 2019. Esophagogastroduodenoscopy (EGD) revealed a lesion in the esophagus, about 20-26cm from the incisor. Biopsy of the lesion was positive for esophageal squamous cell carcinoma (ESCC). The combined positive score (CPS) of programmed cell death ligand 1 (PD-L1) was 2 by DAKO 22C3 and 3 by VENTANA SP263. The patient’s baseline examination showed no abnormalities, including brain MRI (**Figure 1**), and the functional tests of thyroid, heart, lung, liver, and kidney. The patient was hospitalized and received a course of definitive radiotherapy to the primary tumor and regional lymph nodes to a dose of 60 Gy in 30 fractions concurrently with a chemotherapy regimen of paclitaxel and cisplatin. The patient developed severe myelosuppression during the course of chemoradiation therapy. Subsequently, he received four 3-week cycles of consolidative chemotherapy with fluorouracil and cisplatin. The last chemotherapy was received on July 5^th^, 2019.

**Figure 2:**
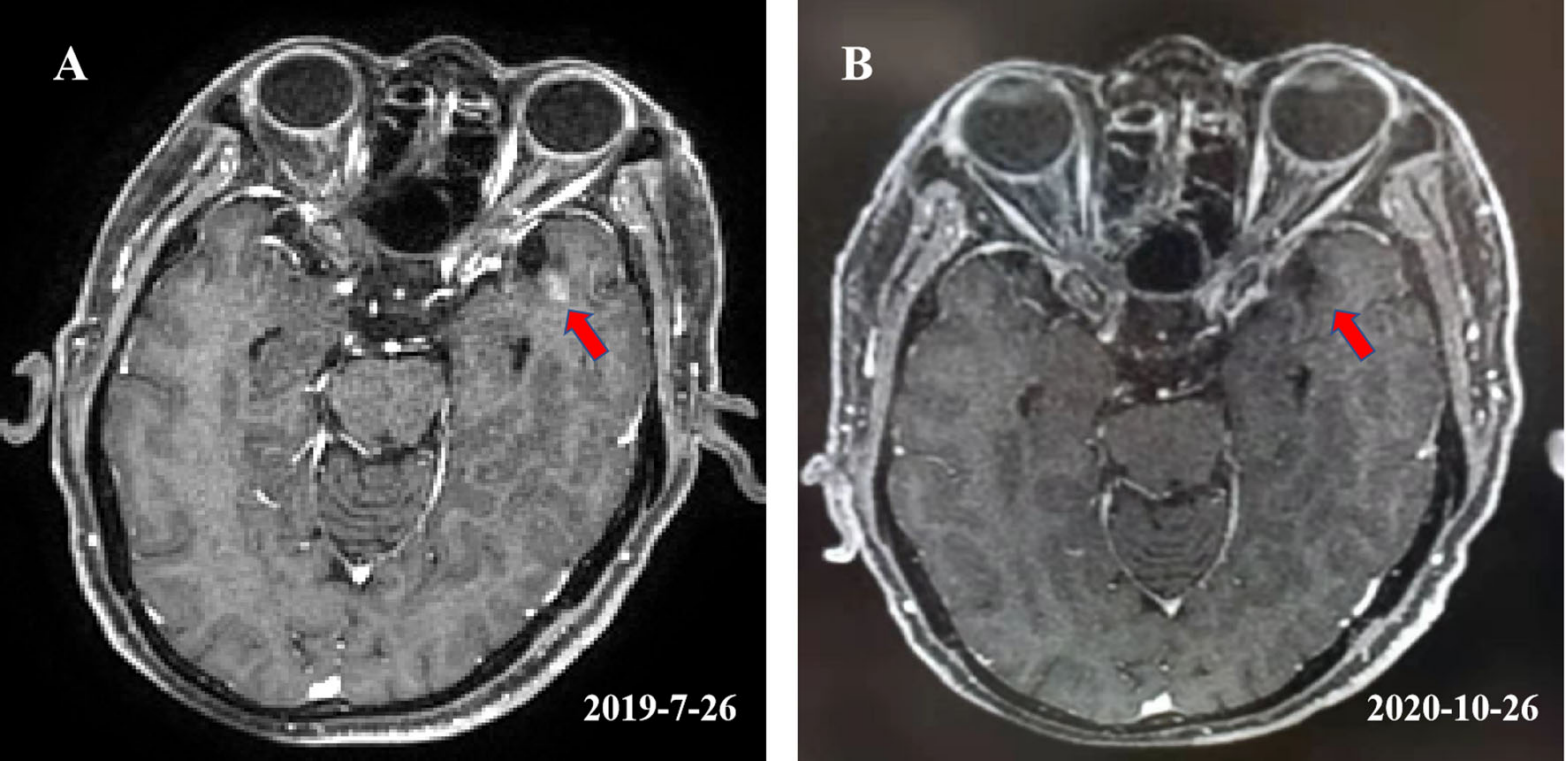
**(A)** Showed a well-circumscribed enhancing nodular lesion with a maximum diameter of about 0.5 cm in the left temporal lobe. **(B)** Showed that the lesion was replaced by a necrotic area after receiving 15 cycles of toripalimab.

On July 26^th^, 2019, the patient returned for follow up. Thoracic computed tomography (CT) showed that the tumor in the esophagus was stable. The patient reported no odynophagia. Unfortunately, the magnetic resonance imaging (MRI) of the brain revealed a new well-circumscribed enhancing nodular lesion in the left temporal lobe (**Figure 2**). Given that his ESCC was positive for PD-L1, toripalimab, one of ICIs, was recommended for his asymptomatic solitary brain metastasis. From August 2019 to October 2020, he received 15 3-week cycles of toripalimab. A follow-up brain MRI revealed that the brain metastasis was replaced by a necrotic area (**Figure 2**). No decline of cognitive function [mini-mental state examination (MMSE) and Loewenstein-cognitive function assessment (LOTCA)] or other acute toxicity have been found during the treatment. Physical examination, brain MRI, esophageal barium swallow, gastroscope, chest radiography, or CT from the neck to the upper abdomen were performed in the follow up. By the last revision of this article, the patient was still on toripalimab with a stable disease at the primary site as well as a good cognitive function.

## Discussion

In this article, we report a case of asymptomatic brain metastasis from EC, which is very rare in itself. We found that the brain metastatic lesion had a complete response to ICIs and the lesion was replaced by a necrotic area after 15 cycles of ICIs. In addition, he is doing generally well with stable disease and good cognitive function. Due to the low incidence of brain metastasis in EC, it is difficult to conduct clinical trials in these patients. Therefore, this case report will add to the existing publications and provide supporting data for using ICIs to treat EC patients with brain metastases, especially for patients with asymptomatic brain metastases.

Surgery is a highly effective treatment for patients with symptomatic brain metastases, especially for patients with a solitary lesion, a good KPS score and a controlled extracranial tumor. Stereotactic radiosurgery (SRS) is also a standard treatment for patients with symptomatic brain oligometastases. Whole-brain radiation therapy (WBRT) is often used for patients who is not suitable for surgery or SRS, such as with multiple brain metastases, leptomeningeal disease or poor performance status. Systemic chemotherapeutic drugs have a limited therapeutic efficacy for brain metastases due to poor penetration of the blood–brain barrier. Targeted therapy has shown some value in certain tumors with brain metastases; for example, osimertinib in epidermal growth factor receptor (EGFR)-mutant non-small cell lung cancer (NSCLC) patients with brain metastases (10, 11) and alectinib in anaplastic lymphoma kinase (ALK)-rearrangements NSCLC patients with brain metastases (12). ICIs have also shown some preliminary evidence, mainly in brain metastases for patients with lung cancer, melanoma and renal cell carcinoma (13, 14).

In recent years, a variety of ICIs have entered the field of oncotherapy and have quickly reshaped management strategies for many types of cancers, such as NSCLC, SCLC, melanoma, lymphoma, esophagus cancer, breast cancer, renal cancer, liver cancer, and urothelium cancer. However, patients with active or untreated brain metastases usually are excluded from clinical trials (15, 16). Although some studies have included patients with stable brain metastases, specific subgroup analyses have not been reported. At present, the research on the ICIs of brain metastases have been mainly focused on melanoma (14, 17–19). The results of these studies demonstrated that ICIs, especially ipilimumab have therapeutic activity in some patients with brain metastases, particularly in patients with small and asymptomatic metastases. The rate of intracranial responses was approximately 20%. In addition, some studies showed that double-drug combined ICI regimens may bring more benefits to these patients. In the study of CheckMate 204, for melanoma patients with asymptomatic untreated brain metastases, the rate of intracranial responses was 57% in patients treated with nivolumab and ipilimumab (14). A multicenter open-label randomized phase 2 trial demonstrated that nivolumab combined with ipilimumab could bring a higher rate of intracranial responses (46% vs. 20%) for patients with asymptomatic untreated brain metastases comparing with nivolumab alone (19). There are a few reports on brain metastasis from lung cancer treated with ICIs. A study from Italy analyzed the efficacy of nivolumab in 409 patients with asymptomatic, neurologically stable brain metastases from non-squamous non-small cell lung cancer. The results showed that the objective response rate (ORR) and the disease control rate (DCR) were 17% and 39% in these patients, with similar toxicity rates in the overall study population with or without brain metastases (9). An exploratory analysis of a phase III OAK study showed that atezolizumab could prolong the median OS (16.0 vs 11.9 months) of patients with asymptomatic, treated brain metastases compared with docetaxel (20). Activating the immune system is the mechanisms by which ICIs kill tumor cells, by which blood-brain barrier could not block.

Brain radiotherapy was an important treatment options for patients with brain metastases. Preclinical findings have suggested that the combined application of brain radiotherapy and ICIs could yield synergistic effects, bringing more benefits to patients (21, 22), while clinical studies on ICIs combined with radiotherapy are very limited, particularly in patients with brain metastases. Notably, many prospective trials often removed these patients with active brain metastases (23, 24). With respect to brain radiotherapy and ICIs in combination, favorable outcomes have been demonstrated in a few retrospective studies (25–27). The study of Pike et al. reported that brain radiation following the start of PD-1 inhibitors could benefit patients (26). Kotecha et al. evaluated the outcomes of 150 patients who received ICIs and SRS and demonstrated that concurrent radiotherapy and ICIs will maximize the benefits (27). Ahmed et al. found that delivery of brain radiotherapy prior to or during administration of PD-1/PD-L1 inhibitors would bring more survival benefits for patients compared to patients who received brain radiotherapy after ICIs (p=0.006) (25). The ability of concurrent radiotherapy and ICIs to improve patients’ survival may be due to the fact that concurrent ICIs increased the durability of intracranial control that radiotherapy provided. In light of the controversy and limited data, further research on how to combine brain radiotherapy and ICIs are warranted to improve the management strategy for these patients.

On the other hand, with this combined approach, the question we need to ask would be: will the risk of brain radiotherapy-associated adverse events (AEs), especially, radiation necrosis, increase synchronously when combined with ICIs? Radiation necrosis can be a very serious complication for patients who received radiotherapy to brain metastases and may even be life-threatening (28). Combination of SRS and WBRT would increase the incidence of radiation necrosis of brain compared to SRS alone (29). Du Four et al. reported that three patients with brain metastases from melanoma developed radiation necrosis of brain following brain radiotherapy and ipilimumab for the first time (30). Martin et al. found that addition of ICIs would increase the incidence of symptomatic radiation necrosis in patients that underwent stereotactic radiation for brain metastases, especially in patients with melanoma (31). In this study, 23 of 115 (20%) developed symptomatic necrosis in patients with ICIs, significantly higher than 25 of 365 (6.8%) in patients without ICIs. However, the available results are not always consistent. Hubbeling et al. investigated the safety of brain radiotherapy in NSCLC patients receiving PD-1/PD-L1 inhibitors and found that brain radiotherapy and ICIs in combination was not associated with an increase in brain-radiotherapy-associated AEs (32). A great challenge in current oncology practice is to achieve the best possible balance between benefits and adverse events of treatments. In the past, patients with brain metastases usually had a short survival time and had no chance to develop late complications from brain irradiation, such as radiation necrosis of the brain. In the era of immunotherapy, the survival time of these patients have been extended, which have exposed patients to a chance for more late adverse events.

In this study, we report a case of asymptomatic brain metastasis from EC, which had a complete response after 15 cycles of ICIs. The patient since has had stable disease at the primary site and good cognitive function. Similar results have been reported in the past. Flippot et al. had reported the first study assessing the activity of nivolumab in patients with asymptomatic brain metastases from metastatic clear cell renal cell carcinoma (8). In their study, there were 4 out of 34 patients that achieved intracranial complete response after treatment of nivolumab without radiotherapy. Notably, the longest diameter of the lesion in the four patients was less than 10 mm at baseline, which was similar to the patient in our report. Crinò et al. also reported a similar phenomenon in a patient with non-squamous NSCLC, who had two metastatic nodular lesions in the brain (9). After 3 years of treatment with nivolumab without radiotherapy, both metastases had disappeared. These studies suggest that for patients with limited intracranial tumor burden, ICIs may be a sufficient treatment for intracranial tumor control.

These thought-provoking study results invoke us to ask: could ICIs replace surgery or radiation therapy for treating a brain metastatic lesion? The answer was obviously: no. However, the safety profile of ICIs alone appears acceptable and ICIs seemed to control certain brain metastases, especially small and asymptomatic ones. Therefore, ICIs seemed to be appropriate to replace surgery or radiation therapy for patients with small, stable, and asymptomatic brain metastatic lesions. In the meantime, for patients with symptomatic brain metastases, brain surgery and radiotherapy are still the main treatment options. It is worthwhile to note that the use of corticosteroids may affect the efficacy of ICIs. A combination of ICIs and brain radiotherapy may bring favorable outcomes for patients, but we need to pay attention to potentially increased neurologic toxicities caused by this combined therapy.

In conclusion, for patients with asymptomatic brain metastasis, ICIs seem to be a favorable treatment option, while the best treatment options for patients with symptomatic brain metastases remains an open question. More clinical trials of ICIs in patients with brain metastases are warranted.

## Data Availability Statement

The original contributions presented in the study are included in the article/supplementary material. Further inquiries can be directed to the corresponding author.

## Ethics Statement

Written informed consent was obtained from the individual(s) for the publication of any potentially identifiable images or data included in this article.

## Author Contributions

Conceptualization, JW. Data curation, LX and YW. Formal analysis, LX, YL, and JW. Resources, LX, YW, and YL. Validation, CL and JW. Writing-original draft, LX. Writing-review & editing, CL and JW. All authors contributed to the article and approved the submitted version.

## Conflict of Interest

The authors declare that the research was conducted in the absence of any commercial or financial relationships that could be construed as a potential conflict of interest.

## Publisher’s Note

All claims expressed in this article are solely those of the authors and do not necessarily represent those of their affiliated organizations, or those of the publisher, the editors and the reviewers. Any product that may be evaluated in this article, or claim that may be made by its manufacturer, is not guaranteed or endorsed by the publisher.
